# Targeting CD38 with Daratumumab Plus Chemotherapy for Patients with Advanced-Stage Plasmablastoid Large B-Cell Lymphoma

**DOI:** 10.3390/jcm11164928

**Published:** 2022-08-22

**Authors:** Yun Kyoung Ryu, Edd C. Ricker, Craig R. Soderquist, Mark A. Francescone, Andrew H. Lipsky, Jennifer E. Amengual

**Affiliations:** 1 Division of Hematology-Oncology, Department of Medicine, Columbia University Irving Medical Center, New York, NY 10032, USA; 2 Columbia University Vagelos College of Physicians and Surgeons, New York, NY 10032, USA; 3 Department of Pathology, Columbia University Irving Medical Center, New York, NY 10032, USA; 4 Department of Radiology, Columbia University Irving Medical Center, New York, NY 10032, USA

**Keywords:** plasmablastoid, lymphoma, daratumumab, anti-CD38

## Abstract

Plasmablastic lymphoma (PBL) is a rare and aggressive form of large B-cell lymphoma (LBCL) most commonly seen in the setting of chronic immunosuppression or autoimmune disease. The prognosis is poor and CHOP-like regimens often fail to produce durable remission; therefore, there is no established standard of care treatment. However, PBL demonstrates substantial morphologic and immunophenotypic overlap with multiple myeloma (MM), suggesting that MM therapeutics might prove useful in treating PBL. We studied the effects of treatment using the first-in-class monoclonal antibody directed against CD38, daratumumab, in combination with chemotherapy in seven patients with advanced-stage LBCL with plasmablastic features. Treatment was safe and well-tolerated. Among six evaluable patients, six patients had complete response after treatment, and four patients who met strict WHO criteria for PBL had durable response (12–31 months and ongoing).

## 1. Introduction

Plasmablastic lymphoma (PBL) is a rare and aggressive form of large B-cell lymphoma (LBCL) most commonly seen in the setting of chronic immunosuppression, such as HIV infection and organ transplantation, or in patients with pre-existing lymphoproliferative or autoimmune disorders [1,2,3,4,5]. PBL commonly presents at extranodal sites, including the oral cavity, digestive tract and skin, at an advanced stage (III/IV) and in the presence of B symptoms [6]. The prognosis of patients with PBL is generally poor, with a median overall survival of 8 months [7]. Therapeutic strategies to target this disease are limited, as CHOP-like regimens have failed to produce durable remission, and no standard of care has been established [6].

PBL is believed to originate from the plasmablast and generally demonstrates either plasmablastic or immunoblastic morphology, occasionally with plasmacytic differentiation. The immunophenotype is similar to that of plasma cells, with the expression of CD38, CD138 and IRF4/MUM1 and the lack of CD20, PAX5 and BCL-6 [8,9]. Although the pathophysiology of these tumors remains incompletely understood, their emergence is associated with Epstein–Barr virus (EBV) infection in 70% of cases [7], suggesting that pro-survival signals mediated by EBV-encoded proteins may contribute to PBL pathogenesis [6]. Translocations involving *MYC* have also been described in PBL and have been associated with worse survival [4,7,10]. c-MYC is normally expressed in a subset of centrocytes and marks cells preparing to exit the germinal center [11]. The acquisition of a plasmablast phenotype from GC B cells involves the BLIMP1-mediated repression of c-MYC [12]. Whether the translocation of *MYC* in PBL overrides this differentiation network and arrests cells in a plasmablast-like state is unknown.

Daratumumab (Darzalex, Janssen Biotech) is a first-in-class monoclonal antibody directed against CD38 [13] and is approved by the U.S. Food and Drug Administration (FDA) for the treatment of newly diagnosed and relapsed/refractory multiple myeloma (MM). Daratumumab is approved in combination with lenalidomide and dexamethasone (D-Rd) in newly diagnosed patients as per the MAIA trial with the disease-free survival of 70.6% at 30 months for D-Rd vs. 55.6% in Rd [14] and in relapsed/refractory MM as per the POLLUX trial with the overall response rate (ORR) of 92.9% in DR-d compared to 76.4% in the Rd and a higher CR of 43.1% vs. 19% in Rd [15]. Daratumumab is also approved in combination with bortezomib and dexamethasone (DVd) as per the CASTOR trial with the ORR of 82.9% in the daratumumab group compared to 63.2% in the control group [16]. Daratumumab works via several mechanisms, including antibody-dependent cellular cytotoxicity (ATCC), antibody-dependent cellular phagocytosis (ADCP), complement-dependent cytotoxicity (CDC), direct apoptosis and immunomodulation [17,18]. The side-effect profile is well established, with common side effects including infusion related reactions. More important are the cytopenias and infections associated with daratumumab, with one study reporting a total of 11.7% of patients requiring hospitalizations due to infections in 171 patients treated with daratumumab with and without chemotherapy [19]. However, these infections were generally manageable with antivirals and antibiotics. The success of daratumumab in MM patients and its safety profile in combination with chemotherapy suggests that it may be a safe and effective therapeutic option for PBL, which is characterized by high surface CD38 expression. The concept of combining daratumumab plus EPOCH chemotherapy has been proposed by the AIDS Malignancy Consortium for HIV patients with PBL. Here, we report a single-center experience of the treatment of seven patients with advanced-stage LBLC with plasmablastic features using daratumumab in combination with chemotherapy.

## 2. Materials and Methods

**Figure 1:**
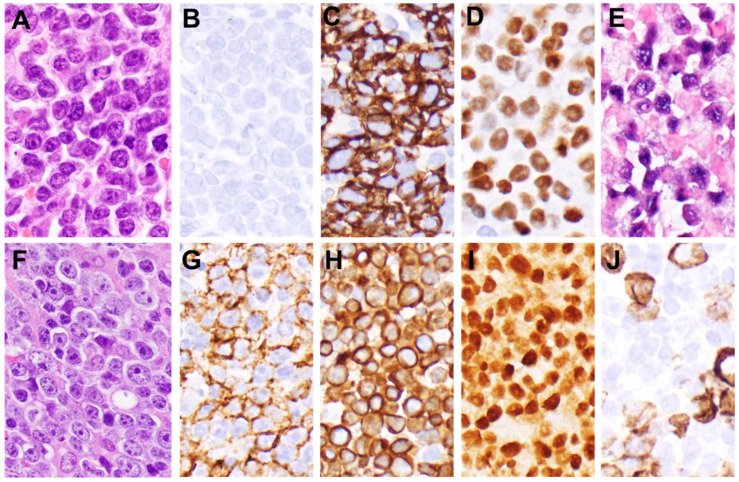
Morphologic and immunophenotypic features of plasmablastic lymphomas. (**A**) A hematoxylin and eosin (H&E)-stained section stained using hematoxylin and eosin (H&E) from case 5 shows plasmablasts which are negative for (**B**) CD20 and diffusely positive for (**C**) CD138. The cells show high expression of (**D**) MYC secondary to an IGH/MYC translocation and show evidence of EBV infection by (**E**) in situ hybridization for EBER. (**F**) An H&E-stained section of case 4 shows large cells with immunoblastic morphology, which are partially positive for (**G**) CD20, diffusely positive for (**H**) CD79a and (**I**) MUM1. (**J**) CD138 is focally positive.

We performed a single-center retrospective analysis of adult patients with LBCL with plasmablastic features or plasmablastic lymphoma treated at Columbia University Irving Medical Center between August 2019 and July 2022. Institutional review board approval was obtained, and the study was performed in accordance with the ethical principles of the Declaration of Helsinki. Inclusion criteria required morphologic and/or immunophenotypic evidence of plasmablastic differentiation. We included five cases of typical plasmablastic lymphoma as defined by the current World Health Organization classification, which expressed plasma cell markers CD138 and IRF4/MUM1 and lacked the B-cell marker CD20. We also included one case of LBCL with plasmablastic features and CD20 positivity and one case of HHV8+ LBCL with plasmablastic features (Figure 1). All biopsies were reviewed by hematopathologists at our institution. The Kaplan–Meier curve was used to calculate progression-free survival and overall survival. Progression-free survival, best response to treatment, duration of response and overall survival were the primary outcome parameters assessed in the study. Progression-free survival was defined as the time from diagnosis until progression of disease (radiographically assessed and confirmed with biopsy). The date of biopsy was defined as the date of diagnosis. Overall survival was defined as the time from diagnosis until death.

## 3. Results

### 3.1. Patient Characteristics

We identified seven patients over 2.5 years with CD38 and/or CD138 expression with plasmablastic lymphoma or LBCL with plasmablastic features who were administered daratumumab in combination with chemotherapy who we analyzed for this manuscript. Detailed demographics of the study population are reported in Table 1. Patient age ranged from 25 to 88 years old, and five of the seven patients were men. All patients had stage 3 disease or higher at the time of diagnosis, and all patients had extranodal sites of disease, including one patient (case 5) with testicular and CNS involvement (Table 1). Four of the seven patients had ECOG scores of 3 or 4 at diagnosis. Four of the seven patients had HIV/AIDS, and two of the seven patients were immunocompromised from a history of organ transplantation or autoimmune diseases on biologics. Six of the seven patients lacked expression of CD19 and CD20 but had high expression of MUM1 and either CD138 or CD38 expression (Table 2). CD38 was tested in five of the seven patients and was positive in all five patients, and CD138 was positive in six of the seven patients. Two patients (case 4 and 7) did not meet the strict criteria for PBL based on the WHO classification. One patient (case 4) demonstrated a large-cell lymphoma with immunoblastic morphology and the expression of CD19, CD20, MUM1, CD38 and partial CD138, most consistent with LBCL with plasmablastic features. One patient (case 7) had a plasmablastic neoplasm expressing MUM1, CD38 and HHV8 but lacking CD20 and CD138, most consistent with an HHV8+ large B-cell lymphoma with plasmablastic features.

### 3.2. Patient Outcomes

**Figure 2:**
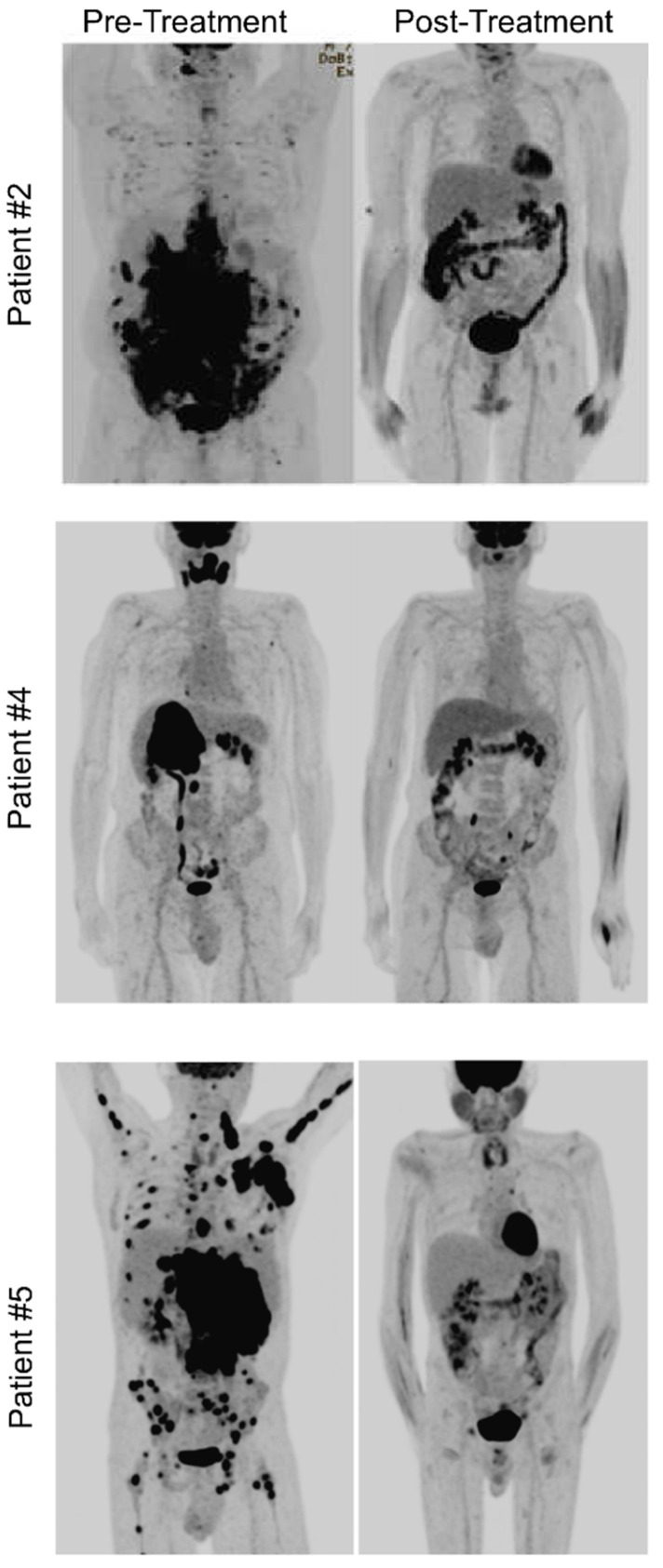
PET-CT of patients’ pre-treatment and post treatment. Patient 5 was thought to have PR at post-treatment, with residual R partially necrotic inguinal lymph node (2.3 × 1.9 cm, SUV max 4.5), but subsequent R inguinal lymph node biopsy demonstrated only a hernia sac and no evidence of lymphoma.

**Figure 3:**
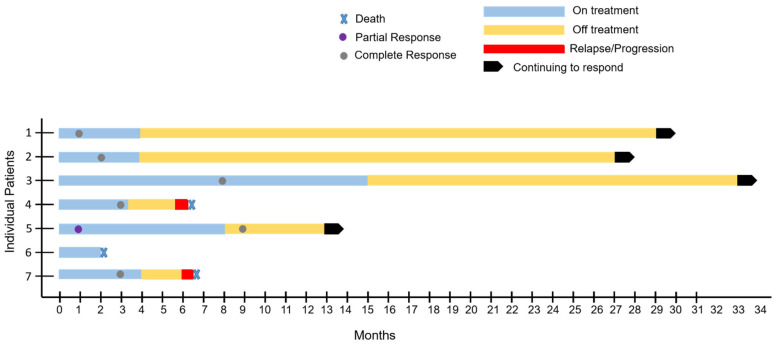
Swimmer plot of patients treated with daratumumab-based regimen. All 7 patients achieved CR with treatment, and 4 patients who have completed treatment are continuing to be in remission.

**Figure 4:**
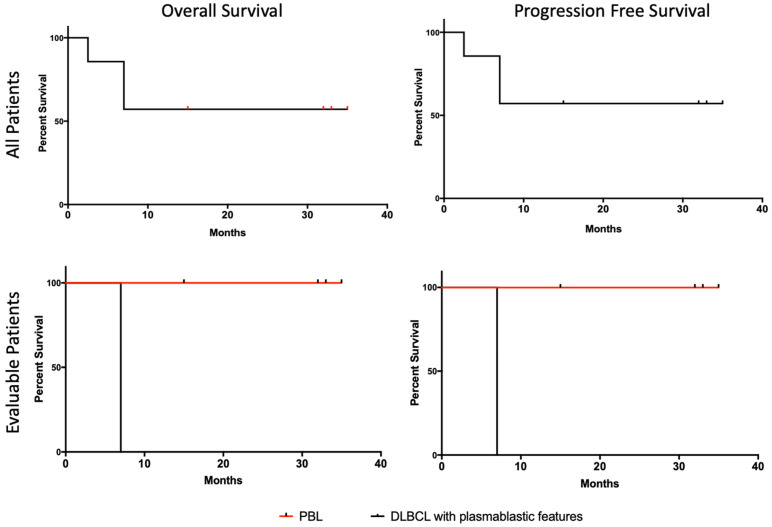
Overall survival and progression-free survival.

Details of treatment course are presented in Table 3. Of the six evaluable patients (cases 1–5 and 7), five patients received six cycles of daratumumab with dose-adjusted EPOCH. One patient with LBCL with plasmablastic features with CD20+ also received six cycles of rituximab (case 4). One patient (case 3) refused chemotherapy due to potential toxicity and initially received four cycles of weekly daratumumab as a single agent, followed by four cycles of weekly bortezomib plus daratumumab, which led to a mixed response. The patient experienced progression of disease and was therefore transitioned to daratumumab plus liposomal doxorubicin, lenalidomide and dexamethasone. The patient obtained a complete remission (CR) at 36 weeks of treatment which was maintained for another year until treatment was halted due to COVID-19 infection. One patient (case 5) with testicular involvement had a left orchiectomy with contralateral radiation following the completion of chemotherapy. This patient also had CNS involvement at diagnosis and, in addition to daratumumab–EPOCH plus intrathecal methotrexate, was treated with alternating high-dose methotrexate. No patients received consolidative autologous stem-cell transplant. Of the six evaluable patients, all patients obtained a CR (100%) (Figure 2). One patient was not evaluable due to non-cancer- and non-treatment-related death. At the time of the data cut-off, the overall median duration of response in the six evaluable patients was 16.8 months and ongoing and was longer in the four evaluable classic PBL patients than in the two who did not meet the strict criteria for PBL (23.7 months and ongoing vs. 3 months) (Figure 3). In the four patients with classic PBL, extended survival meant that progression-free survival (PFS) was not reached; in the other two patients, PFS was 6 months, and both of these patients relapsed quickly upon the completion of therapy and died despite attempts at salvage therapy. Similarly, overall survival (OS) was not reached in the PBL patients (except for case 6, who died from illicit drug use, asthma exacerbation and respiratory arrest); the OS in the other two patients was 7 months (Figure 4). At 24 months, the OS was 57% across all patients, including the patient that died of non-treatment- and non-disease-related causes.

Overall treatment was well-tolerated, and there were no treatment-related deaths. All patients receiving cyclophosphamide had dose reductions for initial cycles given their immunocompromised state or low CD4 count. Cyclophosphamide dose was up-titrated in subsequent cycles. Several patients had the etoposide, doxorubicin and vincristine dose reduced for the initial cycle (cases 1, 4 and 5). Vincristine was omitted in all cycles in case 1 due to lymphoma bowel involvement and surgery prior to the initiation of treatment. Across seven patients, there were six grade-3/4 events. Two patients had grade-3 peripheral neuropathy requiring dose reduction (case 5) or omission of vincristine (case 2). Two patients had grade-3 neutropenic fever requiring hospitalization (one during treatment (case 4) and the other at 2 months after the completion of treatment (case 1)). One patient (case 5) had a persistent fever requiring treatment delay. This patient had severe infections prior to the initiation of therapy and had difficulty clearing them once treatment was initiated. Two patients had grade-3 infections requiring admissions (one with RSV and parainfluenza infection complicated by COPD exacerbation (case 6), and the other with pre-existing Clostridium difficile and norovirus infections occurring at the time of new HIV and plasmablastic lymphoma diagnoses complicated by chronic diarrhea, malnutrition and electrolyte imbalance requiring treatment with nitazoxanide (case 5)). One patient (case 6) had a reaction to daratumumab infusion which included rash and pruritis with cycle 1 requiring the addition of corticosteroids and diphenhydramine. One patient (case 1) had bradycardia with the first daratumumab infusion. One patient (case 1) had PICC-associated DVT complicated by PE requiring thrombectomy and anticoagulation, and another patient (case 5) had incidental findings of PE requiring anticoagulation. One patient (case 1) had high-EBV viremia requiring a dose of rituximab. Treatment was delayed in one patient due to poor compliance and follow up (case 6).

## 4. Discussion

PBL is a rare and aggressive subtype of LBCL that frequently involves extranodal sites and is most often associated with immune deficiency. With most patients presenting advanced-stage disease and a lack of established treatment, the prognosis is generally poor. Here, we report the successful use of daratumumab, a monoclonal antibody directed against CD38, for the treatment of seven patients with plasmablastic lymphoma and LBCL with plasmablastic features.

The median OS in our study was either consistent with, or higher than, previous reports (6–19 months) [4,20,21,22]. A review examining both a single-center series of 25 cases and a meta-analysis of 277 cases reported a median OS of 8 months with considerable variation (0–105 months); there was no significant correlation between the median OS and HIV status (10 months in HIV+ and 11 months in HIV-), or in AIDS vs. post-transplant immunosuppression (7 months) [7,20]. In two systematic reviews of patients with PBL, the median OS was 15 months in 112 HIV+ patients and 9 months in 76 HIV- patients [21,23]. The response rate was higher in our study than reported studies involving treatment with chemotherapy alone or in combination with rituximab. In the LYSA series, of 135 PBL patients, 80% received CHOP, CHOP-like or anthracycline-based regimens such as EPOCH or COPADM or ACVBP; 18% received rituximab and 4% received bortezomib and anthracycline. Seven percent of patients had a dose reduction. CR was reported in 55% of patients, PR in 5%, stable disease in 2% and progressive disease in 19%; mortality was 53% at the 49-month follow up (68% due to lymphoma progression) and the median OS was 32 months [23]. In another multicenter retrospective case series of 50 HIV+ PBL patients from 13 institutions, 80% received chemotherapy (63% with CHOP and 37% with more intensive regimen); the median PFS was 6 months, and the median OS was 11 months, with no difference between CHOP and more intensive regimens. CR was reported in 66% of patients, PR in 5% and no response in 29%^4^. In a German study of 18 HIV+ patients with PBL treated with CHOP or high-dose methotrexate-based B-cell ALL protocols, the median OS was 5 months [24]. The AIDS Malignancy Consortium conducted a 10-year retrospective evaluation of 10 patients receiving chemotherapy; CR was reported in 58%, PR in 17% and refractory disease in 8%; at 1 year, survival was 66.7% [25].

Beyond standard chemotherapy for PBL, MM drugs such as bortezomib and lenalidomide have demonstrated their effectiveness. In a retrospective multicenter analysis of 16 patients with PBL treated with frontline bortezomib-EPOCH, CR was demonstrated in 15/16 patients (95%) and PR in 1/16 patients. The median OS was 62 months, with a 5-year survival rate of 63%. Two patients with CR underwent autologous stem-cell transplantation (ASCT) in the first remission. Four patients (including one who had undergone ASCT) relapsed within 2 years. One patient underwent salvage therapy with ifosfamide, carboplatin and etoposide in combination with daratumumab followed by ASCT and achieved CR [26]. In a retrospective multicenter study of 19 PBL patients who received first-line therapy with bortezomib (in combination with EPOCH or, less often, CHOP, VCD, VAD or VD), complete response was reported in 58%, PR in 16% and no response in 26%. Three-year survival was 59% [27]. Finally, the combination of lenalidomide and the anti-PD1 monoclonal antibody tislelizumab achieved CR in a single PBL patient who was refractory to mini-CHOP and bortezomib [28].

None of the patients in our series reported significant adverse events following administration, despite high ECOG and IPI scores. No treatment-related deaths were observed. There was one death due to respiratory failure in a patient who was not evaluable for response (a polysubstance drug user) that was unlikely to be related to treatment. Overall, treatment was well-tolerated, suggesting that the role of daratumumab for the treatment of PBL should be examined more broadly. Response was independent of the risk factors of PBL such as HIV status, organ transplant and autoimmune disorder. However, given the difference in the response rate of treatment depending on the entity of diseases (PBL vs. LBCL with plasmablastic features), detailed pathologic characterization may be critical, and further studies are warranted to understand the molecular features that may better define the population that will benefit most from DARA-EPOCH.

PBL has a strong association with HIV infection and immunodeficiency, including that caused by autoimmune disorders, transplantation and EBV infections. The estimated incidence of HIV associated PBL is 2% of all HIV-related lymphomas [29]. Currently, there is an open clinical trial studying daratumumab in combination with dose-adjusted EPOCH (DA-EPOCH) in newly diagnosed stage II-IV PBL by the AIDS Malignancy Consortium with the primary objective of evaluating the feasibility of adding daratumumab to DA-EPOCH (NCT04139304). Secondary outcomes will include CR, PFS, OS and adverse events. The inclusion criteria include both HIV-positive and HIV-negative patients with CD4 ≥100 cell/mL, ECOG ≤ 2 and with adequate kidney, cardiac and liver functions. There are no preliminary data from the study available at this time. Of the patients we studied, four of the seven patients would not have met eligibility criteria due to high ECOG, and three of the four HIV-positive patients would have not qualified due to low CD4 counts (<100 cells/mL). Yet, all patients discussed above tolerated and responded well to daratumumab. We believe our findings support the ongoing clinical trial.

Several recent studies performing whole-exome sequencings have identified the disruption of several key regulatory pathways including JAK-STAT3, RAS-MAPK and non-canonical NF-kB signaling in PBL [30]. Gain-of-function JAK/STAT signaling was found in 64% of PBL cases, while RTK-RAS signaling and NF-kB signaling were found in 76% and 58% of patients, respectively. Interestingly, *STAT3* and *LNP1* mutations were significantly more common in HIV+ compared to HIV- patients with PBL, while HIV- patients harbored *TP53*, *PRDM1* and *IRS4* mutations. There also appears to be a difference in mutational landscape based on the EBV status of PBL patients, with EBV- patients having higher mutational and copy-number loads and more frequent *TP53*, *MYC* and *CARD11* mutations and EBV+ patients having more mutations that disrupt the JAK-STAT pathway [31]. MYC status had no correlation with OS or PFS and no bearing on the number of mutations [30]. Furthermore, there was no correlation between *MYC* rearrangement and EBV status [30]. Comparing the mutational signature of PBL with DLBCL, there were three common candidate drivers (*TP53*, *PRDM1* and *HIST1H1E*) and five common genes (*PIM1*, *BTG1*, *CD79B*, *ETS1* and *STAT3*). In addition, there is one common gene between PBL and MM (*NRAS*) [30].

With a better understanding of the mutational landscape of PBL, several potential new targets have been hypothesized including pan-TRK inhibitors such as larotrectinib or entrectinib for *NTRK3* mutations or BTK inhibitors such as ibrutinib in the setting of *BTK* mutations and common gain-of-function of NF-kB signaling [30]. In addition to evaluating these new targeted agents for PBL, other successful strategies that have been used in the setting of MM such as CAR-T and bispecifics against CD38, CD138 or B-cell-maturation antigen (BCMA) are possible options to be considered especially for those with relapsed and refractory PBL.

In summary, a daratumumab-based chemotherapy treatment strategy was well-tolerated and achieved favorable response rates in our patients with PBL. Daratumumab-based chemotherapy regimens should be studied further in an effort to improve outcomes as there is no standard of care. Data from clinical trials such as that by the AIDS Malignancy Consortium will be helpful in delineating the optimal utilization of this type of combination.

## Figures and Tables

**Table 1:** Demographics.

Demographic Feature	N (%)
Age (y), median (range)	48 (25–88)
**Sex (%)**	
Male	71% (5/7)
Female	29% (2/7)
**Race (%)**	
Black	29% (2/7)
White	71% (5/7)
**Ethnicity (%)**	
Hispanic	28.6% (2/7)
Non-Hispanic	71.4% (5/7)
**IPI score**	
ECOG	
0–2	43% (3/7)
3–4	57% (4/7)
Stage	
1–2	0%
3–4	100% (7/7)
Median LDH at diagnosis (range)	277 (228–1817)
Extranodal sites of disease	100% (7/7)
% IPI 1–2	29% (2/7)
% IPI ≥3	71% (5/7)
% of stage 1–2	0% (0/7)
% of stage ≥3	100% (7/7)
**Underlying risks for lymphoma**	
HIV/AIDS	57% (4/7)
Transplant/immunosuppression	29% (2/7)
EBV/CMV	71% (5/7)
Median CD4 count at diagnosis (range) cells/mL	34 (8–184)
CD4 count <100 at diagnosis	75% (3/4)
Median % of CD4 cells	6% (2–25%)
Neoplastic markers	
CD20+	14% (1/7)
CD19+	14% (1/7)
CD38+	100% (5/5)
CD138+	86% (6/7)
MUM1+	100% (7/7)
c-myc+	100% (7/7)

**Table 2:** Disease Characteristics.

Patient	Age	Sex	Race	Risk for Lymphoma	Treatments	Duration of Response	Best Response	Notable Toxicity	Pathology
1	28	F	White	Heart transplant/PTLD/EBV viremia	6 cycles of low dose Dara-EP(O)CH 1 dose of ritux	27 mo ongoing	CR after c2	PICC associated DVT + PE s/p thrombectomy Neutropenic fever ×1 episode 2 months after completion of chemotherapy	CD138+/−
									CD38+
									MUM1+
									C-Myc > 90%
									CD20−
									EBER−
									Ki67 80%
									Kappa lambda−
									P53+/−
									Neg for IgH gene arrangement
2	73	M	African American	HIV/AIDS CMV, EBV	6 cycles of EPOCH- Dara added on c2 (1 dose c2, 2 doses from C3–6) Vincristine omitted on C6	25 mo ongong	CR at interim scan after 4 cycles	Neuropathy- held vincristine on C6	CD138++
									MUM1++
									BCL6−
									c-MYC+
									BCL2+/−
									CD79a+/−
									CD20−
									ki67 80–90%
3	88	F	White	Chron’s disease on infliximab	dara × 4 weekly mixed response- > bortezomib 1.3 mg/m^2^ + dara × 4 weekly PD- > dara + lenalidomide + doxil × 36 weeks	21 mon ongoing	CR	None	Variable CD138+/−
									MUM1++
									EBER ISH+
									CD79a+
									CD10++
									CD20-
									Ki 67 70–80%
									c-Myc+/−
									BCL2−
4	79	M	White	None	6 cycles DARA- R-EPOCH alternating with IT MTX At relapse R-DHAX- Daratumumab	4/2021–7/2021 so 3 mo	CR after 4 cycles of DARA-R EPOCH	Neutropenic fever × 1 episode	Sparsely CD138+, CD38+
									CD19+, CD20+/−
									CD79a+
									MUM1+
									BCL6dim+
									BCL2+/0
									C-Myc+/−
									CD10−
									Kappa−
									Lambda+
									Ki67 70–80%
									EBV neg
									Foxp1+
									45,X,Y,ins(3;1)(p21.1;p13q33),del(1)(q41q42),del(6)(q16q24),t(14;15)(q32;q11.2),i(17)(q10)][19]/46,XY
									IgH gene rearrangement+
5	48	M	Hispanic	HIV/AIDS (CD4 of 4 VL 191K) EBV	Dara-EPOCH × 6 cycles LP with HD MTX × 3 cycles Dara- HD MTX × 2		PR after 2 cycles	Peripheral neuropathy Anemia requiring transfusion Difficulty clearing pre-exisiting infection (i.e., Norovirus causing critical hypoK and hypoMg from chronic diarrhea and malnutrition requiring treatment with nitazoxadine)	CD138++,
									CD38 bright
									P53+
									MUM1+
									C-MYC 40%
									CD30+/0
									CD20− CD79a−
									BCL6−, BCL2−, diffuse CD38
									Positive for EBV
									Ki67 > 80%
									Myc rearrangement in 93%
6	43	M	White	HIV (off treatment, VL 7576 at dx) CMV, EBV	DARA-EPOCH × 3 cycles with IT MTX		unevaluable	COPD exacerbation from parainfluenza infection requiring supplemental oxygen and steroid	MUM1++
									CD138+/−
									CD38+
									BCL2+/−
									BCL6−
									CD79a+/−
									C-Myc+
									P53+/−
									CD30−
									Ki67 high
									CD20-
									EBERISH+/−
7	34	M	African American	HIV/AIDS (CD4 of 11, VL > 100K) EBV	Dara-EPOCH × 6 cycles alternating with IT-MTX		CR after 4 cycles		MUM++
									CD138−
									CD38+/−
									MUM1+
									CD45+/0
									Ki67 80–90%
									P53+ minor subset
									CD20−, CD19−, c-myc+/−,
									76~84<3N>, XXY, add(1)(q21), +2, +3, add(3)(p21), +7, +8, +12, +12, del(12)(q14q24.1)x1~2, hsr(12)(q24)x3, +16, +17, +19, +20, −22, −22[cp5]/46,XY [17]IgH gene rearrangement

**Table 3:** Treatment course and detailed doses of daratumumab-based regimen. Five patients competed 6 cycles of Dara-R-EPOCH based regimen. One patient was not able to complete the treatment due to non-treatment-related and non-disease-related death. One patient declined chemotherapy due to concern for possible toxicity and received daratumumab with bortezomib followed by liposomal doxorubicin and lenalidomide.

Patient	Treatment Course						
1	C1 of Dara-EPOCHDoxorubicin 7 mg/m^2^ Etoposide 30 mg/m^2^ Cyclophosphamide 187 mg/m^2^ Dara 16 mg/kg on weekly throughout between cycles	C2 of Dara-EPOCH Doxorubicin 7 mg/m^2^ Etoposide 37 mg/m^2^ Cyclophosphamide 377 mg/m^2^ Dara 16 mg/kg on weekly throughout between cycles	C3 of Dara-EPOCH Doxorubicin 10 mg/m^2^ Etoposide 50 mg/m^2^ Cyclophosphamide 400 mg/m^2^ Rituximab 550 mg Dara 16 mg/kg on weekly throughout between cycles	C4 of Dara-EPOCH Doxorubicin 10 mg/m^2^ Etoposide 50 mg/m^2^ Cyclophosphamide 562 mg/m^2^ Dara 16 mg/kg on D1	C5 of Dara-EPOCH Doxorubicin 7 mg/m^2^ Etoposide 50 mg/m^2^ Cyclophosphamide 364 mg/m^2^ Dara 16 mg/kg on D4 HD Methotrexate 3.5 g/m^2^	C6 of Dara-EPOCH Doxorubicin 7 mg/m^2^ Etoposide 40 mg/m^2^ Cyclophosphamide 242 mg/m^2^ Dara 16 mg/kg on d4	
2	C1 of EPOCH Doxorubicin 10 mg/m^2^ Etoposide 50 mg/m^2^ Vincristine 0.4 mg/m^2^ Cyclophosphamide 375 mg/m^2^	C2 of Dara-EPOCH Doxorubicin 10 mg/m^2^ Etoposide 50 mg/m^2^ Vincristine 0.4 mg/m^2^ Cyclophosphamide 350 mg/m^2^ Dara 16 mg/kg on D1	C3 of Dara-EPOCH Doxorubicin 10 mg/m^2^ Etoposide 50 mg/m^2^ Vincristine 0.4 mg/m^2^ Cyclophosphamide 750 mg/m^2^ Dara 16 mg/kg on D1	C4 of Dara-EPOCH Doxorubicin 10 mg/m^2^ Etoposide 50 mg/m^2^ Vincristine 0.4 mg/m^2^ Cyclophosphamide 750 mg/m^2^ Dara 16 mg/kg on D1 and D5	C5 of Dara-EPOCH Doxorubicin 10 mg/m^2^ Etoposide 50 mg /m^2^ Vincristine 0.4 mg/m^2^ Cyclophosphamide 750 mg/m^2^ Dara 16 mg/kg on D1 and D5	C6 of Dara-EPOCH Doxorubicin 7.5 mg/m^2^ Etoposide 37.5 mg/m^2^ Cyclophosphamide 562 mg/m^2^ Dara 16 mg/kg on D1 and D5	
3	Dara 1.6 mg/kg weekly × 4	Bortezomib 1.3 mg/m^2^ weekly × 4Dara 16 mg/kg weekly × 4	Dara 16 mg/kg q2 weekly Liposomal doxorubicin 40 mg/m^2^ q2 weekly Revlimid 15 mg daily or 20 mg q2 days				
4	C1 of Dara-R- EPOCH Vincristine 0.4 mg/m^2^ Doxorubicin 8 mg/m^2^ Etoposide 40 mg/m^2^ Cyclophosphamide 600 mg/m^2^ Rituximab 375 mg/m^2^ Dara 16 mg/kg on D1 and D5 Inthrathecal MTX 12 mg	C2 of Dara-R- EPOCH Vincristine 0.8 mg/m^2^ Doxorubicin 15 mg/m^2^ Etoposide 75 mg/m^2^ Cyclophosphamide 600 mg/m^2^ Rituximab 375 mg/m^2^ Dara 16 mg/kg on D1 and D5 Inthrathecal MTX 12 mg	C3 of Dara-R- EPOCH Vincristine 0.8 mg/m^2^ Doxorubicin 15 mg/m^2^ Etoposide 75 mg/m^2^ Cyclophosphamide 600 mg/m^2^ Rituximab 375 mg/m^2^ Dara 16 mg/kg on D1 and D5 Inthrathecal MTX 12 mg	C4 of Dara-R- EPOCH Vincristine 0.8 mg/m^2^ Doxorubicin 15 mg/m^2^ Etoposide 75 mg/m^2^ Cyclophosphamide 600 mg/m^2^ Rituximab 375 mg/m^2^ Dara 16 mg/kg on D1 and D5 Inthrathecal MTX 12 mg	C5 of Dara-R- EPOCH Vincristine 0.4 mg/m^2^ Doxorubicin 10 mg/m^2^ Etoposide 50 mg/m^2^ Cyclophosphamide 375 mg/m^2^ Rituximab 375 mg/m^2^ Dara 16 mg/kg on D1 and D5 Inthrathecal MTX 12 mg	C6 of Dara-R- EPOCH Vincristine 0.4 mg/m^2^ Doxorubicin 10 mg/m^2^ Etoposide 50 mg/m^2^ Cyclophosphamide 375 mg/m^2^ Rituximab 375 mg/m^2^ Dara 16 mg/kg on D1 and D5 Inthrathecal MTX 12 mg	C1 of R-DHAX Oxaliplatin 180 mg D1 Cytarabine 1130 mg D2 and D3
5	C1 of Dara-EPOCH Doxorubicin 10 mg/m^2^ Etoposide 37.5 mg/m^2^ Vincristine 0.3 mg/m^2^ Cyclophosphamide 187.5 mg/m^2^ Dara 16 mg/kg on D1 and D8 Intrathecal MTX 12 mg C1D15 HD MTX 6 g/m^2^ with Dara 16 mg/kg	C2 of Dara-EPOCH Doxorubicin 10 mg/m^2^ Etoposide 37.5 mg/m^2^ Vincristine 0.2 mg/m^2^ Cyclophosphamide 187.5 mg/m^2^ Dara 16 mg/kg on D1 Intrathecal MTX 12 mg	C3 of Dara-EPOCH Doxorubicin 10 mg/m^2^ Etoposide 50 mg/m^2^ Vincristine 0.4 mg/m^2^ Cyclophosphamide 187.5 mg/m^2^ Dara 16 mg/kg on D1 Intrathecal MTX 12 mg C3D15 HD MTX 6 g/m^2^ with Dara 16 mg/kg	C4 of Dara-EPOCH Doxorubicin 10 mg/m^2^ Etoposide 50 mg/m^2^ Vincristine 0.2 mg/m^2^ Cyclophosphamide 187.5 mg/m^2^ Dara 16 mg/kg on D1 Intrathecal MTX 12 mg	C5 of Dara-EPOCH Doxorubicin 10 mg/m^2^ Etoposide 50 mg/m^2^ Vincristine 0.4 mg/m^2^ Cyclophosphamide 187.5 mg/m^2^ Dara 16 mg/kg on D1 Intrathecal MTX 12 mg C5D15 HD MTX 6 g/m^2^ with Dara 16 mg/kg	C6 of Dara-EPOCH Doxorubicin 10 mg/m^2^ Etoposide 50 mg/m^2^ Vincristine 0.4 mg/m^2^ Cyclophosphamide 187.5 mg/m^2^ Dara 16 mg/kg on D1 Intrathecal MTX 12 mg	
6	C1 of Dara-EPOCH Doxorubicin 10 mg/m^2^ Etoposide 50 mg/m^2^ Vincristine 0.4 mg/m^2^ Cyclophosphamide 375 mg/m^2^ Dara 16 mg/kg on D1 Intrathecal MTX 12 mg	C2 of Dara-EPOCH Doxorubicin 10 mg/m^2^ Etoposide 50 mg/m^2^ Vincristine 0.4 mg/m^2^ Cyclophosphamide 375 mg/m^2^ Dara 16 mg/kg on D1 Intrathecal MTX 12 mg	C3 of Dara-EPOCH Doxorubicin 12 mg/m^2^ Etoposide 60 mg/m^2^ Vincristine 0.4 mg/m^2^ Cyclophosphamide 900 mg/m^2^ Dara 16 mg/kg on D1 and D5				
7	C1 of Dara-EPOCH Doxorubicin 10 mg/m^2^ Etoposide 50 mg/m^2^ Vincristine 0.4 mg/m^2^ Cyclophosphamide 187 mg/m^2^ Dara 16 mg/kg on D1 Intrathecal MTX 12 mg	C2 of Dara-EPOCH Doxorubicin 10 mg/m^2^ Etoposide 50 mg/m^2^ Vincristine 0.4 mg/m^2^ Cyclophosphamide 350 mg/m^2^ Dara 16 mg/kg on D1 and D5 Intrathecal MTX 12 mg	C3 of Dara-EPOCH Doxorubicin 10 mg/m^2^ Etoposide 50 mg/m^2^ Vincristine 0.4 mg/m^2^ Cyclophosphamide 350 mg/m^2^ Dara 16 mg/kg on D1 and D5 Intrathecal MTX 12 mg	C4 of Dara-EPOCH Doxorubicin 10 mg/m^2^ Etoposide 50 mg/m^2^ Vincristine 0.4 mg/m^2^ Cyclophosphamide 350 mg/m^2^ Dara 16 mg/kg on D1 and D5 Intrathecal MTX 12 mg	C5 of Dara-EPOCH Doxorubicin 10 mg/m^2^ Etoposide 50 mg/m^2^ Vincristine 0.4 mg/m^2^ Cyclophosphamide 350 mg/m^2^ Dara 16 mg/kg on D1 and D5 Intrathecal MTX 12 mg	C6 of Dara-EPOCH Vincristine 0.4 mg/m^2^ Doxorubicin 10 mg/m^2^ Etoposide 50 mg Vincristine 0.4 mg/m^2^ Cyclophosphamide 350 mg/m^2^ Dara 16 mg/kg on D1 and D5 Intrathecal MTX 12 mg	Bortezomib 1.5 mgC1 of DHAX Bortezomib 2.1 mgOxaliplatin 210 mg Cytarabin 2100 mg on D1 and D2 C2 of DHAX Bortezomib 2.8 mgOxaliplatin 275 mg Cytarabine 4200 mg on D1 and D2

## Data Availability

Not applicable.
